# PDGF Modulates BMP2‐Induced Osteogenesis in Periosteal Progenitor Cells

**DOI:** 10.1002/jbm4.10127

**Published:** 2019-01-15

**Authors:** Xi Wang, Brya G Matthews, Jungeun Yu, Sanja Novak, Danka Grcevic, Archana Sanjay, Ivo Kalajzic

**Affiliations:** ^1^ Department of Reconstructive Sciences UConn Health Farmington CT USA; ^2^ Department of Orthopedic Surgery UConn Health Farmington CT USA; ^3^ Department of Physiology and Immunology School of Medicine University of Zagreb Zagreb Croatia; ^4^ Department of Molecular Medicine and Pathology University of Auckland Auckland New Zealand

**Keywords:** PERIOSTEUM, BONE MORPHOGENETIC PROTEIN 2, PLATELET‐DERIVED GROWTH FACTOR, STEM CELLS, FRACTURE HEALING

## Abstract

BMPs are used in various clinical applications to promote bone formation. The limited success of the BMPs in clinical settings and supraphysiological doses required for their effects prompted us to evaluate the influence of other signaling molecules, specifically platelet‐derived growth factor (PDGF) on BMP2‐induced osteogenesis. Periosteal cells make a major contribution to fracture healing. We detected broad expression of PDGF receptor beta (PDGFRβ) within the intact periosteum and healing callus during fracture repair. In vitro, periosteum‐derived progenitor cells were highly responsive to PDGF as demonstrated by increased proliferation and decreased apoptosis. However, PDGF blocked BMP2‐induced osteogenesis by inhibiting the canonical BMP2/Smad pathway and downstream target gene expression. This effect is mediated via PDGFRβ and involves ERK1/2 MAPK and PI3K/AKT signaling pathways. Therapeutic targeting of the PDGFRβ pathway in periosteum‐mediated bone repair might have profound implications in the treatment of bone disease in the future. © 2018 The Authors *JBMR Plus* is published by Wiley Periodicals, Inc. on behalf of the American Society for Bone and Mineral Research.

## Introduction

Fracture repair involves complex interactions between cell lineages under the spatiotemporal control of growth factors and cytokines.^1^, ^2^ Understanding the mechanisms that regulate commitment and differentiation of mesenchymal progenitor cells is critical for the advancement of regenerative medicine. Bone marrow stromal cells (BMSCs) have been well‐characterized over the years as an experimental model for osteoblast precursors and other mesenchymal lineages.^3^ However, the periosteum, which harbors progenitor cells, plays an essential role during fracture healing.^4^, ^5^ Previous studies have shown that murine periosteum‐derived progenitor cells (PDCs) are able to differentiate into chondrogenic, osteogenic, and adipogenic lineages in vitro, and promote angiogenesis.^6^, ^7^ Removal of the periosteum severely impairs bone healing and successful bone graft incorporation requires contribution from the periosteum.^4^, ^8^ Therefore, it is critical to understand the growth factor and signaling mechanisms that occur in PDCs following injury.

Platelet‐derived growth factor (PDGF) and its receptors are highly expressed in a variety of cell types during fracture healing.^9^, ^10^ PDGF, which is released by platelets and macrophages at fracture sites during the initial inflammation stage, is highly mitogenic, chemotactic, and enhances angiogenesis. There are five members of the PDGF family: PDGF‐AA, AB, BB, CC, and DD. The effects of PDGF are mediated through receptor tyrosine kinases PDGFRα and PDGFRβ, which can form both homodimers and heterodimers.^9^ PDGF‐BB is considered the most universal because it can activate all three PDGFR isoforms. Although PDGF has been approved for limited clinical applications,^11^ its effects on osteogenic differentiation and bone formation are not fully understood. The response to PDGF is cell‐type dependent; to date, the effect of PDGF on periosteal cells has not been studied.

Bone morphogenetic protein 2 (BMP2) expression in mesenchymal cells is essential for the initiation of fracture healing and the osteogenic and chondrogenic differentiation of periosteal progenitors in bone graft transplantation experiments.^12^, ^13^ Recombinant human BMPs (BMP2 and BMP7) are approved for use in spinal fusion and the treatment of open tibia fractures.^14^ The BMPs potently induce osteogenic differentiation in vitro and in vivo; however, supraphysiological doses of BMP2 are required for clinical use, causing deleterious side‐effects and high costs.^15^, ^16^

Whereas canonical BMP2‐Smad signaling regulates osteogenic differentiation, other pathways such as growth factor‐activated receptor tyrosine kinases may interfere and dampen its signaling and effects.^17^ In this study, we aimed to evaluate the effects of PDGF/PDGFRβ signaling on the osteogenic differentiation of PDCs, as well as cell proliferation and apoptosis, which are critical for bone regeneration. We discovered an inhibitory effect of PDGF on BMP2‐induced periosteal osteogenesis and investigated the underlying interactions between two pathways.

## Materials and Methods

### Mouse strains

All animal procedures were approved by the institutional animal care and use committee. The Col2.3GFP mice were previously described.^18^ Six‐ to eight‐week C57BL/6 and Col2.3GFP mice were used for cell culture studies and 8‐ to 12‐week‐old female mice were used for fracture studies.

### Fractures

Closed transverse femoral fractures were created on the right femur of 8‐ to 12‐week‐old mice. Prior to surgery, mice were administered 0.1 mg/kg body weight buprenorphine and anesthetized by isoflurane inhalation. A 25G needle (Becton Dickinson, Franklin Lakes, NJ, USA) was inserted in the medullary cavity before fracture to stabilize the fracture. A drop‐weight blunt guillotine device was used to create fracture and the middiaphyseal location was confirmed by X‐ray (Faxitron LX‐60; Faxitron Bioptics, Tucson, AZ, USA). Closed transverse tibial fractures on both tibias were performed for the flow cytometry experiments as described previously.^7^

### Histology

Frozen sections from undecalcified bones were obtained using a tape transfer system.^7^ Immunostaining was performed for PDGFRα (1:80, AF1062, R&D Systems, Minneapolis, MN, USA) and PDGFRβ (1:100, MA5‐15143; Thermo Fisher Scientific, Waltham, MA, USA) as previously described.^19^ Images were acquired on an Axioscan microscope (Carl Zeiss, Thornwood, NY, USA). Following fluorescent imaging, sections were stained with Mayer's hematoxylin (Sigma–Aldrich, St. Louis, MO, USA) or 0.02% Fast green followed by 0.1% Safranin O.

### Isolation of periosteum‐derived cells

Femora and tibiae from 6‐ to 8‐week‐old mice were dissected free of muscle and connective tissue. Epiphyses were then removed and bone marrow was flushed. The periosteum was scraped and enzymatically digested for 1 hour at 37 °C on an orbital shaker (0.5 mg/mL collagenase P, 2 mg/mL hyaluronidase in PBS). Following washing, 2 × 10^4^ cells/cm^2^ were seeded in growth medium (α‐MEM,10% FBS, penicillin streptomycin solution) and cultured in 5% oxygen for the first 4 days. Half medium was changed on day 4 and cultures were then incubated in ambient oxygen. Primary cells reach confluence by day 7 to 8 and were used for differentiation experiments. Passage 1 cells were used for proliferation, apoptosis, Western blots, and immunocytochemistry experiments.

### Flow cytometry and cell sorting

Intact periosteum or calluses pooled from fractured long bones of 2 to 3 mice were isolated to perform cell surface marker analysis as described previously.^7^ Staining was performed using commercially available antibodies (Supplementary Table S1). Cell sorting and analysis was performed using a FACSAria II or LSRII (BD Biosciences, San Jose, CA, USA). Gates were set based on unstained samples, single‐stained controls, and fluorescence minus one (FMO) controls.

### CFU‐F assays

Freshly isolated PDCs were gated on (CD45, Ter119, CD31)^−^ population. These cells were further separated based on the expression of PDGFRα and PDGFRβ. Cells were seeded at a density of 500 cells/well in 6‐well plates in triplicate and maintained in α‐MEM 20% FBS. Cells were cultured in 5% oxygen for 9 to 10 days with half medium replaced on day 4, then fixed in 10% formalin, and stained with crystal violet solution. Crystal‐violet‐stained colonies were counted for assessment of CFU‐F.

### Osteogenic differentiation

Primary PDC were seeded in 12‐well plates and cultured in growth medium for 7 days and then induced towards osteogenic differentiation (50 μg/mL of ascorbic acid, 4mM of β‐glycerophosphate) for another 14 days. Recombinant human BMP2 (Peprotech, Rocky hill, NJ, USA) and recombinant rat PDGF‐BB (R&D Systems) were added from day 7 onwards. Medium with growth factors was changed every 2 days. Col2.3GFP expression was detected using a Zeiss Oberver Z.1 inverted microscope and whole‐well images were scanned for quantification. Cells were stained for alkaline phosphatase (ALP) staining using a commercially available kit (Sigma‐Aldrich) according to the manufacturer's instructions. For CFU‐ALP assay, sorted cells were seeded at density of 2500 cells/well in 12‐well plates, cultured in α‐MEM 20% FBS for 9 days, then osteogenic medium for 14 days.

### Quantitative RT‐PCR

Cells were lysed with Trizol (Thermo Fisher Scientific), and RNA was extracted as per the manufacturer's instructions. 1 μg RNA was treated with DNase (Invitrogen, Carlsbad, CA), cDNA generated using the ImProm‐II Reverse Transcription kit (Promega, San Luis Obispo, CA, USA) and real‐time PCR were performed using TaqMan assays or SYBR green‐based assay (Applied Biosystems, Thermofisher, Waltham, MA, USA; Life Technologies, Grand Island, NY, USA) as previously described.^7^ Primer sequences for the genes examined are shown in Supplementary Table S2.

### Proliferation assay

Passaged PDCs were seeded at a density of 5 × 10^4^ cells/cm^2^ in 6‐well plates and incubated in growth medium for 24 hours. Medium was changed to DMEM and 0.5% FBS; cells were incubated with growth factors overnight. Cells were treated with 10 μM 5‐ethynyl‐2’‐deoxyuridine (EdU) for 4 hours, then harvested by trypsinization and stained with the Click‐It EdU Alexa Fluor 647 cell proliferation assay kit (Invitrogen; Thermo Fisher Scientific) as per the manufacturer's instructions. The number of EdU‐positive cells was determined by flow cytometry and data analysis was performed using DIVA software (Becton Dickinson Biosciences, San Jose, CA, USA).

### TUNEL assay

Passaged PDCs were seeded at a density of 3 × 10^4^ cells/chamber on 8‐chamber slide in 300‐μL growth medium. After 48 hours, they were starved in 0.1% BSA for 72 hours and then treated with 10 ng/mL PDGF‐BB and/or 100 ng/mL BMP2 for 20 hours. After treatment, the Click‐iT plus TUNEL Assay (Alexa Fluor 647) was utilized according to the manufacturer's instructions (Life Technologies; Thermo Fisher Scientific). The cells were counterstained with Hoechst 33342 (Invitrogen; Thermo Fisher Scientific) and TUNEL positive nuclei were counted in ImageJ.

### Western blots

Passaged PDCs were lysed in mRIPA buffer and whole‐cell lysates were electrophoresed on 4–15% gradient (Bio‐Rad, Hercules, CA) or 10% SDS‐PAGE gel according to standard protocols. PVDF membranes were incubated with anti‐phospho‐Smad 1/5/8, anti‐phospho‐ERK1/2, anti‐phospho‐AKT, anti‐phospho‐tyrosine at 1:1000 dilution. The blots were stripped and reprobed with anti‐Smad1, anti‐ERK1/2, anti‐AKT, anti‐PDGFRβ, and anti‐GAPDH (Supplementary Table S3). The amount of protein in individual bands was quantified by ImageJ.

### Immunocytochemistry

Passaged PDCs (3 × 10^4^ cells) were plated on glass coverslips and treated with ERK1/2 inhibitor U0126 (10μM) or PI3K inhibitor LY294002 (10 μM), or DMSO for 1 hour after serum starvation. Cells were then treated with PDGF‐BB (10 ng/mL) and/or BMP2 (100 ng/mL) for 30 min. Coverslips were fixed in 4% paraformaldehyde for 5 min at room temperature, washed in PBS, and blocked in 5% goat serum with 0.3% Triton‐X‐100 for 1 hour. Anti‐pSmad1/5/8 (1:400) was applied overnight. After washing, cells were incubated with goat antirabbit Alexa 488 (1:500; Life Technologies) for 2 hours at room temperature. Cells were counterstained with DAPI and mounted on glass slides prior to imaging on Leica fluorescent microscope (Leica, Wetzlar, Germany). pSMAD positive nuclei were counted for quantification.

### Statistical analysis

Results were analyzed using either Student's *t* test or one‐way ANOVA with Turkey's post hoc test using GraphPad Prism. Data are presented as mean ± SD. *p* values <0.05 were considered statistically significant.

## Results

### Periosteal progenitor cells express PDGFRβ during fracture repair

**Figure 1:**
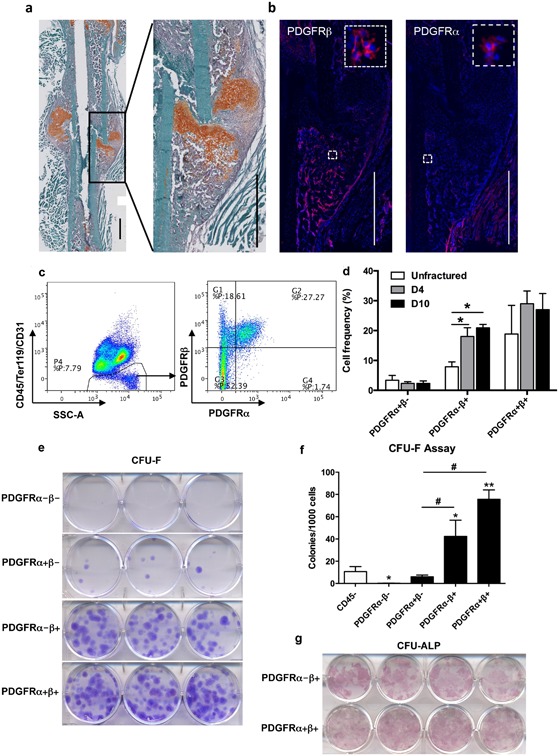
Periosteal progenitor cells express PDGFRβ during fracture repair. (*A,B*) Representative images of PDGFR expression in femurs 10 days post fracture. (*A*) Frozen section of fractured femur was stained with Safranin O/fast green. Callus area was magnified in the box. (*B*) Immunostaining of PDGFRβ and PDGFRα on the same area. Boxed areas show higher magnification. (Red: Alexa Fluor 647; Blue: DAPI). Scale bars = 1000 μm. (*C*) Analysis of the frequency of PDGFR^+^ cells in day 10 periosteal callus by flow cytometry. PDGFR expression was analyzed within (CD45, Ter119, CD31)^−^ population. Representative dot plots of flow cytometry are shown. (*D*) Quantification of cell frequency at different time points following fracture. Both tibias from two mice were pooled for each sample, *n* = 3 samples for each group. (*E*) Representative images of CFU‐F assay. Cells were sorted based on PDGFR expression from (CD45/Ter119/CD31)^−^ population of intact periosteum. Colonies were stained with crystal violet. (*F*) Quantification of the number of CFU‐F colonies from two independent sorting experiments. (*G*) Representative images of CFU‐ALP assay. Sorted cells were induced to osteogenic differentiation for 14 days. Osteoblastic colonies were shown by ALP staining. Values are mean ± SD. *p < 0.05 versus control,**p < 0.01 versus control, **^#^** *p* < 0.01.

Expression of PDGFRs in healthy and injured murine periosteum was evaluated by immunostaining. PDGFRβ expression was detected in periosteum, endosteum, and within bone marrow of intact bone (Supplementary Fig. S1a). Four days after femur fracture, the population of PDGFRβ‐expressing cells increased within the thickened periosteum and in the newly formed callus around the fracture site (Supplementary Fig. S1b). Ten days after fracture, PDGFRβ was broadly expressed within the callus, with the exception of the chondrogenic lineage (Fig. 1 *A*, *B*). We detected fewer cells expressing PDGFRα, with a similar location as PDGFRβ^+^ cells (Fig. 1 *B*). We performed flow cytometry to determine the frequency of cells expressing PDGFRs on isolated periosteal cells. Consistent with histological observation, within the non‐hematopoietic/endothelial lineage populations (CD45/Ter119/CD31)^−^ of intact periosteum, around 30% of cells express either one or both PDGFR(Fig. 1 *C*, *D*). Following fracture, there was a significant increase in the proportion of PDGFRβ^+^ cells. We observed very few PDGFRα^+^β^−^ cells in periosteum or fracture callus, and most PDGFRα^+^ cells coexpressed PDGFRβ (Fig. 1 *D*).

To evaluate progenitor potential, we sorted and assessed the colony‐forming capacity of PDGFR^+^ periosteal cells. PDGFRα^−^β^+^ and α^+^β^+^ populations showed significantly greater colony‐forming potential than both the total CD45^‐^ population and PDGFRβ^‐^ populations, confirming the enrichment of mesenchymal progenitor cells in PDGFRβ^+^ populations (Fig. 1 *E*, *F*). To examine the osteogenic potential of PDGFR‐expressing cells, sorted periosteal cells were induced towards osteogenic differentiation for 14 days. PDGFRβ^−^ cells could not differentiate into osteoblasts, regardless of the expression of PDGFRα (data not shown), but PDGFRβ^+^ cells formed osteoblastic colonies indicated by ALP staining (Fig. 1 *G*).

**Figure 2:**
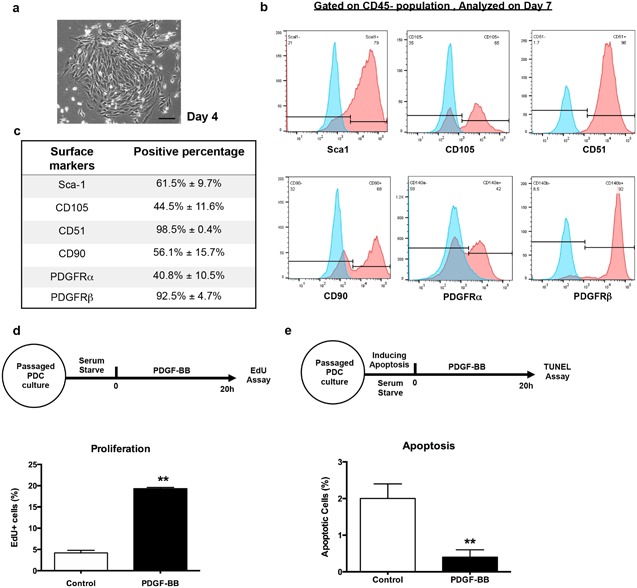
Periosteal progenitor cells express PDGFRβ and respond to PDGF in vitro. (*A*) Representative image of primary periosteum derived cells on day 4. Scale bars = 100 μm. (*B*) Flow analysis of primary PDCs gated on CD45^‐^ nonhematopoietic population. Red histograms represent marker expression; blue histograms represent unstained controls. Shown is the representative data from four independent experiments. (*C*) Summary of flow analysis in (*B*), the percentage of positive cells is shown as the mean ± SD (*n* = 4). (*D*) Passaged PDCs were serum starved overnight and treated with 10 ng/mL PDGF‐BB for 20 hours. Cells were then incubated with EdU for 4 hours and proliferating cells were analyzed by flow cytometry. (*E*) Passaged PDCs were induced to apoptosis by serum starving in 0.1% BSA for 72 hours. Cells were then treated with 10 ng/mL PDGF‐BB for 20 hours. Apoptotic cells were detected by TUNEL assay and counted by positive nuclei. Values are mean ± SD from three independent experiments. **p < 0.01 versus control.

Isolated PDCs grow in colonies in vitro and exhibit fibroblast‐like morphology (Fig. 2A). Flow cytometric analysis on CD45^−^ primary PDCs revealed that the majority of cells express MSC markers: Sca1, CD105, CD51, and CD90 after in vitro expansion (Fig. 2 *B*, *C*) and express PDGFRβ (92.4% ± 4.7%), as well as PDGFRα (40.8% ± 10.5%), the majority of which is coexpressed with PDGFRβ (data not shown).

### PDGF signaling negatively regulates osteogenesis

The high proportion of periosteal cells expressing PDGF receptors (particularly PDGFRβ) indicates responsiveness of PDCs to PDGF. We therefore examined the impact of PDGF‐BB on PDCs in vitro. PDGF treatment resulted in a fourfold increase of cell proliferation (Fig. 2 *D*). In response to serum starvation, apoptosis was also significantly decreased in the presence of PDGF‐BB (Fig. 2 *E*).

**Figure 3:**
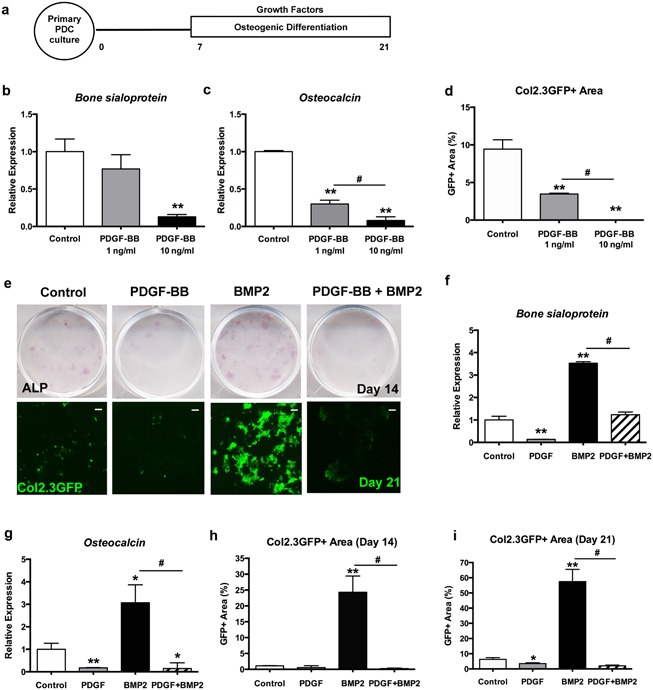
PDGF negatively regulates osteogenesis. (*A*) Experimental design: Primary PDCs isolated from Col2.3GFP mice were cultured for 7 days then induced to osteogenic differentiation with different growth factors for 14 days. (*B*) Gene expression of *bone sialoprotein*, (*C*) *osteocalcin*, and (*D*) quantification of Col2.3GFP area. (*E*) Representative images of ALP staining on day 14 and scans of culture plates showing Col2.3GFP expression in PDCs on day 21. Scale bar = 1000 µm (*F*) Gene expression of *bone sialoprotein*, (*G*) *osteocalcin*, and quantification of Col2.3GFP area on (*H*) day 14 and (*I*) day 21. Values are mean ± SD. *p < 0.05 versus control, **p < 0.01 versus control, **^#^** *p* < 0.01. A representative of three experiments is shown.

The direct effects of PDGF‐BB on osteogenesis have been controversial and cell type‐dependent.^20^ We isolated primary PDCs from Col2.3GFP mice, which harbor a transgene that labels mature osteoblasts.^18^ Col2.3GFP is not detectable in cultures that have not been treated with osteogenic medium. Primary PDCs were induced to osteogenic differentiation in the presence of PDGF‐BB after confluence (Fig. 3 *A*). Upon PDGF‐BB treatment, osteogenic differentiation was inhibited in a dose‐dependent manner indicated by decreased bone sialoprotein and osteocalcin expression and reduced Col2.3GFP+ area (Fig. 3 *B*, *D*).

BMP2 activity is required for periosteal progenitor cell differentiation, which drives bone healing.^13^, ^21^ Therefore, we further examined the combinatory effects of PDGF‐BB and BMP2 on the osteogenic differentiation of PDCs. We chose 10 ng/mL PDGF‐BB in the following study because it has a clear effect on PDC proliferation and differentiation. As expected, BMP2 (100 ng/mL) treatment significantly induced the formation of mature osteoblasts as shown by GFP expression, but this effect was suppressed by treatment with PDGF‐BB (Fig. 3 *E–I*). These data suggest that PDGF‐BB may act as a negative regulator of BMP2 signaling in vitro.

### PDGF‐BB inhibits canonical BMP2‐Smad signaling

**Figure 4:**
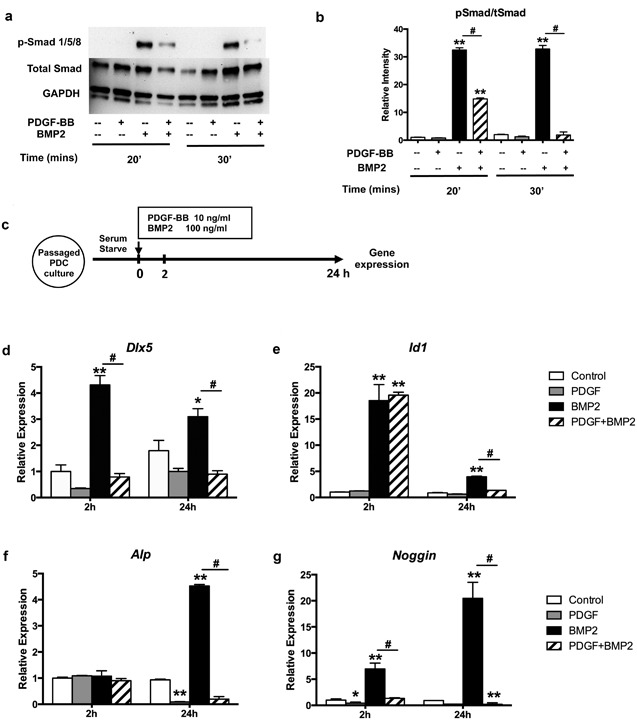
PDGF‐BB inhibits canonical BMP2‐Smad signaling. (*A*) PDCs were treated with 10 ng/mL PDGF‐BB and/or 100 ng/mL BMP2 for 20 or 30 min and cell lysates were separated by SDS‐PAGE and, blots were probed for p‐Smad, total Smad, and GAPDH. The gels presented are representative of three experiments. (*B*) Quantification of Western blot bands intensity. (*C*) Experimental design: passaged PDCs were treated with PDGF‐BB and/or BMP2 for 24 hours after serum starvation. The mRNA level of gene expression was assessed by qRT‐PCR at different time points. (*D–G*) Gene expression of *Dlx5*, *Id1, Alp*, and *Noggin* at 2 and 24 hours after treatment of PDCs. The average expression of untreated control was normalized to 1. Values are mean ± SD. *p < 0.05 versus control, **p < 0.01 versus control, **^#^** *p* < 0.01. A representative of three experiments is shown.

To understand the mechanism underlying the negative regulation of osteogenic differentiation by PDGF‐BB, we investigated its effects on downstream targets of BMP2. We assessed the phosphorylation of Smad1/5/8 in response to BMP2 and PDGF‐BB. Smad1/5/8 was rapidly phosphorylated by BMP2 in PDCs, this effect was reduced in the presence of PDGF‐BB (Fig. 4 *A, B*). Although the expression of endogenous BMP2 and BMP receptors (*Bmpr1a* and *Bmpr2; Bmpr1b* was undetectable) was not affected by PDGF‐BB (Supplementary Fig. S2a–c), expression of BMP target genes *Dlx5* and *Id1* was inhibited upon treatment with PDGF‐BB (Fig. 4 *C–E*). Compared to control, PDGF‐BB significantly inhibited *Alp* expression after 24 hours, whereas BMP2‐induced *Alp* expression was also suppressed by PDGF‐BB (Fig. 4 *F*). Expression of *Noggin*, a BMP2 antagonist was strongly induced by BMP2 and this effect was blocked by PDGF‐BB (Fig. 4 *G*). Together these results indicate inhibitory effects of PDGF‐BB on BMP2 signaling target genes.

**Figure 5:**
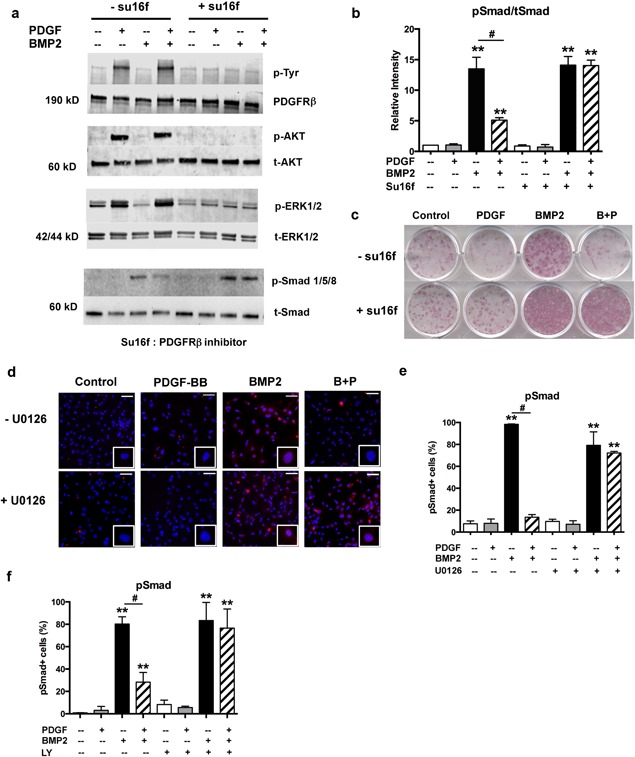
ERK1/2 MAPK and PI3K/AKT pathway is involved in PDGF‐BB/PDGFRβ signaling of PDCs. (*A*) Passaged PDCs were pretreated with PDGFRβ inhibitor su16f (5μM) for 1 hour, followed by 10 ng/mL PDGF‐BB and/or 100 ng/mL BMP2 for 30 min. Total cell lysates were immunoblotted using indicated antibodies. (*B*) Quantification of band intensity. (*C*) Primary PDCs were induced to osteogenic differentiation on day 7 with the absence or presence of su16f (5 μM) for another 14 days. Shown are representative images on day 21. (*D*) Passaged PDCs were pretreated with ERK1/2 inhibitor U0126 (10μM) for 1 hour, followed by growth factors for 30 min. pSmad^+^ cells were detected by immunofluorescence and counterstained with DAPI. Magnified images show positive or negative pSmad staining in nucleus of single cell. Representative images from three independent experiments are shown. Scale bar = 50 µm (*E*) Quantification of pSmad positive cells. (*F*) Passaged PDCs were pretreated with PI3K inhibitor LY294002 (10 μM) for 1 hour, followed by the treatment with growth factors for 30 min. pSmad^+^ cells were detected by immunofluorescence and quantified. LY = LY294002. Values are mean ± SD from three independent experiments. *p < 0.05 versus control, **p < 0.01 versus control, **^#^** *p* < 0.01.

To investigate the crosstalk between PDGF and BMP2 signaling, we examined the downstream pathways in PDCs. PDGF‐BB treatment activated phosphorylation of ERK1/2 and AKT, whereas BMP2 had no effect on these pathways, and did not alter the PDGF‐mediated signal (Supplementary Fig. S3a). Because PDGF‐BB can activate both PDGFRα and PDGFRβ we blocked PDGFRβ signaling using a PDGFRβ inhibitor, su16f.^22^ The inhibition of PDGFRβ signaling blocked PDGF‐BB‐induced phosphorylation of ERK1/2 and AKT, as well as suppressed the effect of PDGF‐BB on BMP2/Smad signaling in PDCs (Fig. 5 *A, B*). As shown by ALP staining, treatment of cultures with PDGFRβ inhibitor eliminated the inhibitory effects of PDGF‐BB on BMP2‐induced osteogenic differentiation (Fig. 5 *C*).

To identify the pathways mediating the effects of PDGF‐BB, we targeted its downstream signaling. The addition of U0126, ERK1/2 inhibitor rescued the phosphorylation of Smad1/5/8 as detected in immunocytochemistry (Fig. 5 *D, E* and Supplementary Fig. S3b). Because no activation of ERK1/2 was induced by BMP2 in PDCs (Supplementary Fig. S3a), the rescue effect of ERK1/2 inhibitor was specific to PDGF signaling. The addition of PI3K inhibitor (LY294002) exhibited similar effects by rescuing inhibitory effects of PDGF‐BB (Fig. 5 *F* and Supplementary Fig. S3c). Thus, at least two downstream signaling mechanisms (ERK1/2 MAPK and PI3K/AKT pathway) are involved in PDGF‐BB signaling that exerts inhibitory effects on BMP2‐induced osteogenesis in PDCs.

## Discussion

It is well‐established that upon injury periosteal reaction is essential for bone regeneration. Characteristics and cellular behavior of PDCs have not been well‐defined.^23^ Although the role of Wnt/β‐catenin, transforming growth factor‐beta (TGF‐β/BMP and Indian hedgehog‐PTHrP, in osteogenic differentiation of periosteal cells have been studied, the synergy or opposing effects on differentiation of progenitors is not understood.^24^, ^25^ PDGFs and their receptors are expressed during the initial phase of fracture healing.^10^ PDGF is released by platelets and serves as the primary initiating signal for cellular ingress. In this study, we demonstrate that PDGF signaling is an important negative regulator of osteogenesis in PDCs in vitro.

The importance of PDGFRβ signaling has been studied during development and it is a potent regulator of mesenchymal stromal cell function.^26^, ^27^, ^28^ In a conditional knock‐in mouse model, Olson and Soriano^29^ found that increased PDGFRβ signaling drives cell proliferation and opposes differentiation of vascular smooth muscle and pericytes, maintaining progenitor potential in vivo. Depletion of PDGFRβ in mesenchymal stromal cells also decreased the proliferation and migration response, while promoting osteogenic differentiation.^30^

In the present study, we observed expression of PDGFRβ in the intact periosteum. The frequency of PDGFRβ^+^ cells doubles after injury in the early periosteum‐derived callus, implicating a major role this pathway might play in the healing process. The importance of PDGFRβ signaling in periosteal progenitor cells is also supported by their colony‐forming potential in vitro. In PDCs compared to PDGFRβ, the abundance of PDGFRα is much lower and the majority of PDGFRα^+^ cells coexpress PDGFRβ. This expression pattern is retained in vitro, suggesting that PDGFRβ is the main path for PDGF signaling in PDCs. Consistent with our findings, in bone marrow stromal cells, Tokunaga and colleagues^30^ showed that PDGF‐AA, which acts via PDGFRα, had no effect on osteogenic differentiation. Upon deletion of the PDGFRβ gene in MSCs, the inhibitory effect of PDGF‐BB on differentiation was abolished, even though PDGFRα expression was increased in PDGFRβ^del/del^ cells. PDGFRα and PDGFRβ are not expressed at the same time and locations during development, and the phenotypes of the knockout mice are also different, suggesting that the expression of these receptors is regulated by different mechanisms.^31^ Based on our in vivo data on expression of PDGF receptors and ability of isolated cell populations to form MSC like colonies, we concluded that PDGFRβ signaling plays a predominant role in PDCs.

As a potent mitogen and chemotactic factor, PDGF promotes the proliferation and migration (data not shown) of PDCs consistent with its effects on other mesenchymal cell types in many studies. In addition, despite its proliferative effect, PDGF‐BB potently inhibits BMP2‐induced osteogenesis of PDCs. Osteogenesis in the absence of added BMP2 was also inhibited consistent with reports in other murine systems.^30^ These observations led us to infer that the inhibitory effect of PDGF on osteogenesis involves modulating BMP2 signaling. We found that PDGF‐BB inhibited canonical BMP2‐Smad signaling, and the expression of BMP2 target genes. This effect was attributable to PDGFRβ because PDGFRβ inhibition rescued the effects of PDGF on signaling and osteogenesis.

We did not observe activation of Smad signaling by PDGF‐BB in PDCs. In addition, we examined the endogenous BMP2 and receptors expression in PDCs. There are two groups of BMP receptors (type I and II) and two subtypes of type I receptor (IA and IB).^32^ It has been shown that both types of receptors are strongly induced in the periosteum in the early stage of fracture repair.^33^, ^34^ Though type IB receptor (*Bmpr1b*) was not detectable in PDCs, the mRNA expression level of *Bmpr1a*, *Bmpr2*, and *Bmp2* was not affected by PDGF‐BB. BMP2 has been reported to activate noncanonical pathways including ERK1/2 and p38 MAPKs in osteogenic cells and cell lines.^35^, ^36^, ^37^ In the present study, we did not detect activation of a noncanonical MAPK pathway in PDCs treated with BMP2, and our data suggest inhibitory effects of ERK MAPK on Smad signaling.

Other studies have reported antagonism of TGFβ/BMP2/Smad signaling by receptor tyrosine kinases with varying mechanisms. Sapkota and colleagues^38^ showed that MAPK catalyzes phosphorylation in the Smad1 linker region, enabling its recognition by ubiquitin ligase Smurf1 leading to the downregulation of BMP2 signaling. PDGF‐BB also induces microRNA‐24 expression, resulting in reduced expression of Tribbles‐like protein‐3 and Smad proteins.^39^

**Figure 6:**
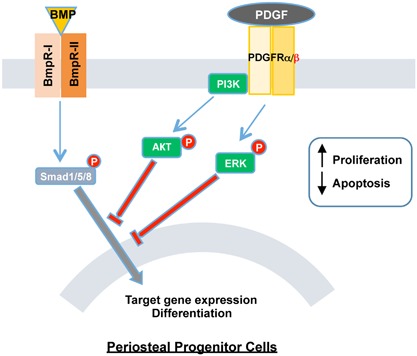
Proposed mechanism of PDGF effects in periosteum derived progenitor cells and its interaction with BMP2/Smad signaling. The binding of PDGF with its receptors, mainly through PDGFRβ, activate multiple signaling (including ERK1/2 MAPK and PI3K/AKT pathways) in PDCs. It promotes the proliferation and prevents the apoptosis of PDCs. During osteogenic differentiation, however, PDGF signaling blocks the canonical BMP2/Smad pathway, inhibiting the target gene expression thus osteogenesis of PDCs.

Here we propose a mechanism underlying the effects of PDGF/PDGFRβ signaling in PDCs (Fig. 6). In response to the injury, there is an increase in the expression of PDGFRβ or expansion of cells expressing PDGFRβ. The binding of PDGF to PDGFRβ activates ERK1/2 MAPK and PI3K/AKT signaling in PDCs, thereby promoting cell proliferation and inhibiting apoptosis. However, during osteogenesis, PDGF‐BB downstream signaling reduces the canonical BMP2/Smad pathway and target gene expression, suppressing cell differentiation while maintaining their proliferative potential. This effect is mediated via both ERK1/2 MAPK and PI3K/AKT signaling pathways. Further studies are required to understand how these interactions play out in vivo.

Our findings may be relevant to clinical settings. Recombinant BMP2 is approved by the FDA for the treatment of fracture repair and spinal fusion.^14^, ^15^ Despite the abundant osteogenic effects in vitro, the effective concentration of rhBMP2 for human use is over 10,000 times higher than that of cultured cells, and in addition to bone formation, can result in harmful inflammation and bone resorption.^14^ It is possible that other growth factors limit the ability of BMP2 to induce bone formation. Considering the stepwise nature of bone repair, BMP2 and PDGF may play distinct roles at different stages of fracture healing. Periosteal progenitor cell recruitment and proliferation, promoted by PDGF, could be essential for the following osteogenic differentiation induced by BMP2. Apart from the role in osteogenesis, PDGF also affects other aspects of fracture healing. PDGF stimulates the production of osteoprotegerin, potentially inhibiting bone resorption.^40^ PDGF‐BB secreted by preosteoclasts also induces angiogenesis during coupling with osteogenesis.^41^ Interestingly, PDGF has also been used to promote bone formation in vivo,^24^ despite the majority of in vitro studies showing inhibitory effects on osteogenic differentiation.^30^, ^42^ Understanding the signaling pathways in the context of fracture healing is therefore important to optimize the use of BMPs; new findings might result in the ability to lower the high doses of BMP2 required for clinical effects. Overall, our study explored the effects of PDGF in PDCs and discovered the crosstalk between PDGF and BMP2 signaling during osteogenic differentiation.

## Disclosures

The authors have nothing to disclose.

## Supporting information

Supporting Data S1.
