# Supplementary material for: PDGF Modulates BMP2‐Induced Osteogenesis in Periosteal Progenitor Cells
Source: JBMR Plus. 2019 Jan 15;3(5):e10127. doi: 10.1002/jbm4.10127 (PMC6524680; doi:10.1002/jbm4.10127)
Supplement: Supplementary file 1 — Supporting Data S1. [file JBM4-3-na-s001.pdf]

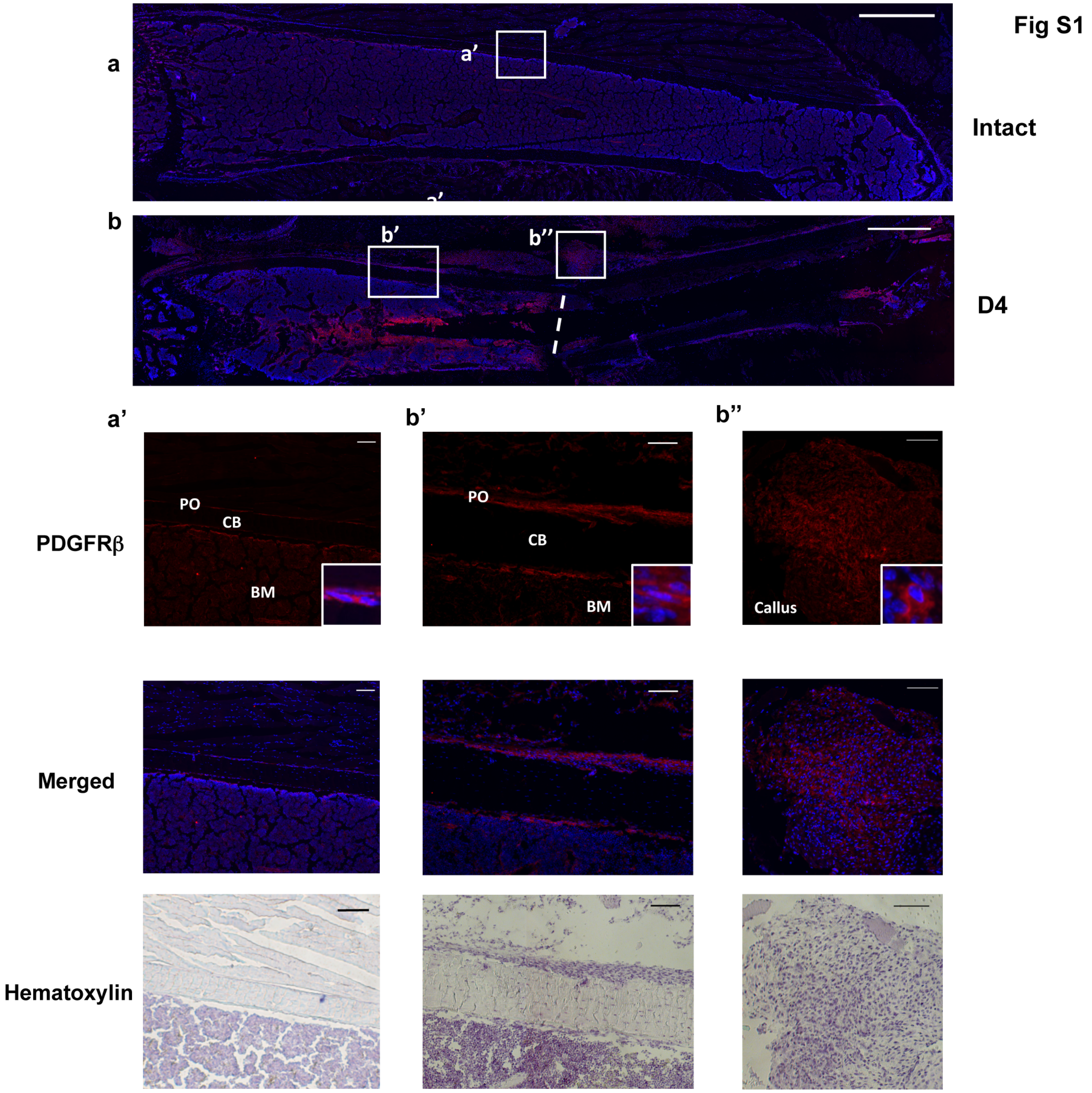

Figure S1: Periosteal progenitor cells express PDGFRβ during fracture repair.
(a). Representative images of PDGFRβ expression in intact periosteum by immunofluorescent staining. Frozen section of intact femur from a 8-week old mouse was stained with anti-PDGFRβ (Red: Alexa Fluor 647) and DAPI (blue). (a’). PDGFRβ cells were detected on periosteum, endosteum and bone marrow and PDGFRβ was expressed on cell surface (magnified image showing representative cells on periosteum). The same section was stained by Hematoxylin.
(b). Representative images of PDGFRβ expression on day 4 after fracture by immunofluorescent staining. Dashed line indicates fracture site. Thickened periosteum close to fracture site (b’) and periosteal callus (b’’) areas were magnified.
PO=periosteum, CB= cortical bone, BM= bone marrow. Scale bars=1000μm (a-b); 100 μm (a’-b’’).

Fig S2

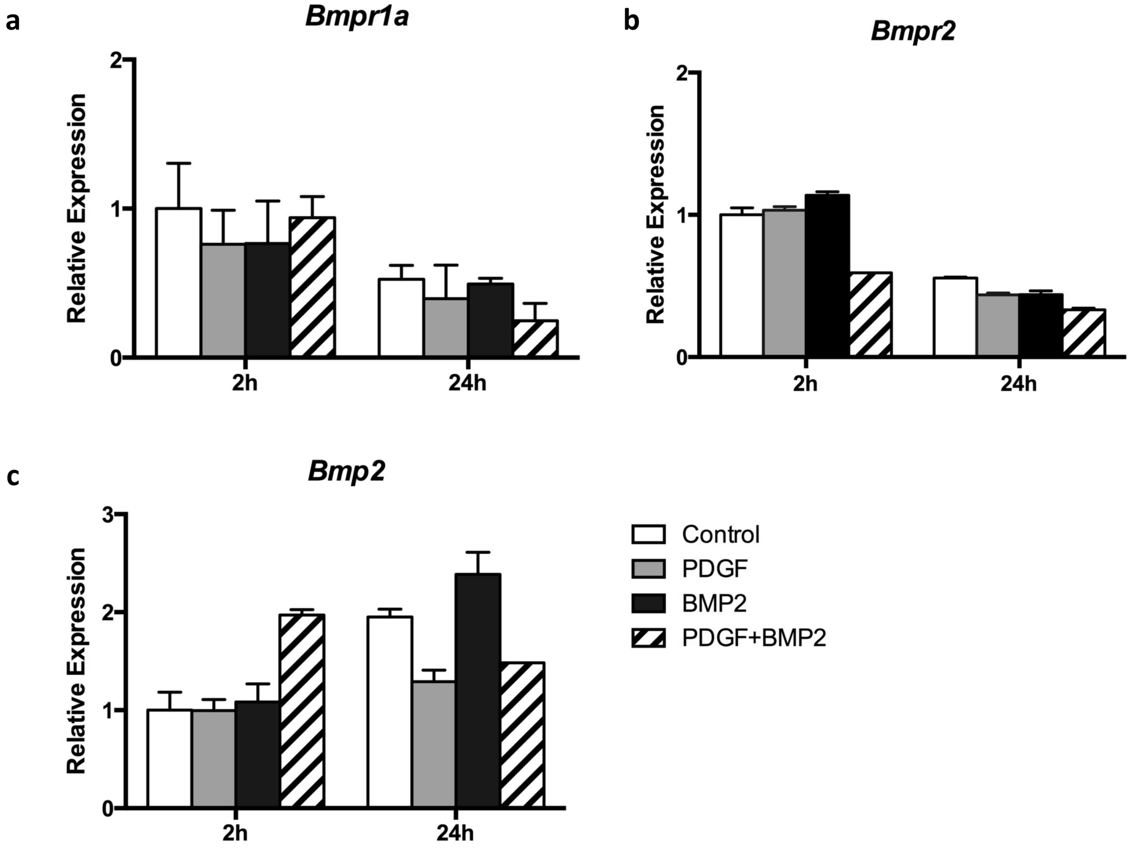

Figure S2: Endogenous BMP2 and BMPR expressions were not changed by PDGF-BB.
PDCs were treated with PDGF-BB and/or BMP2 for 24 hours after serum starvation. The mRNA level of gene expression was assessed by qRT-PCR at different time points. Gene expression of Bmpr1a (a), Bmpr2 (b) and Bmp2 (c) at 2 and 24 hours after treatment of PDCs. The average expression of untreated control was normalized to 1. Values are mean ± SD, A representative of three experiments is shown.

Fig S3

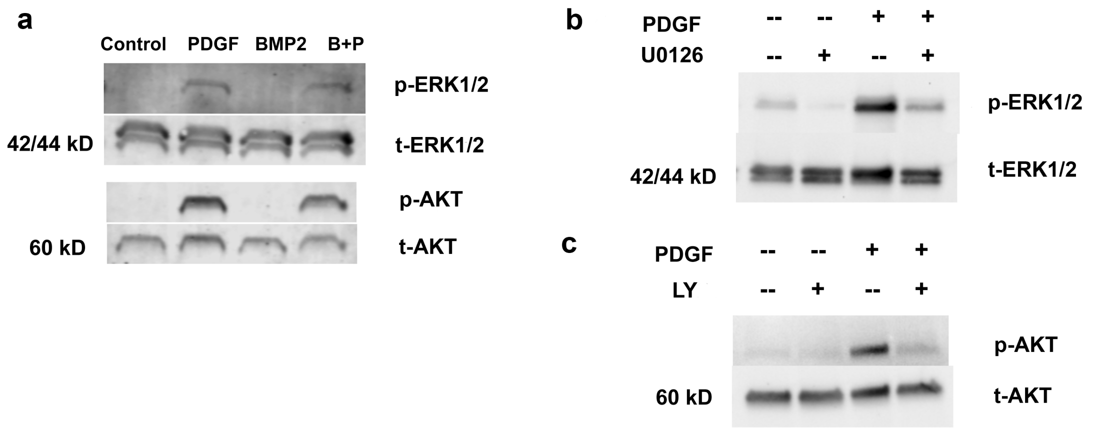

Figure S3: PDGF-BB activated ERK1/2 MAPK and PI3K/AKT pathway in PDCs.
(a). PDCs were treated with 10ng/ml PDGF-BB and/or 100ng/ml BMP2 for 30 minutes. Total cell lysates were immunoblotted with anti-pAKT, anti-AKT anti-pERK1/2 anti-ERK1/2.
(b). PDCs were pretreated with ERK1/2 inhibitor U0126 (10μM) for 1 hour, followed by the treatment of 10ng/ml PDGF-BB for 30 minutes. Total cell lysates were immunoblotted with anti-pERK1/2 and anti-ERK1/2.
(c). PDCs were pretreated with AKT inhibitor LY294002 (10μM) for 1 hour, followed by the treatment of 10ng/ml PDGF-BB for 30 minutes. Total cell lysates were immunoblotted with anti-pAKT and anti-AKT.
LY= LY294002. The gels presented are representative of three experiments.

Table S1: Antibodies used for flow cytometry.

Epitope	Conjugate	Manufacturer	Clone	Dilution
CD45	eFluor 450	eBioscience	30-F11	1:400
Ter119	eFluor 450	eBioscience	TER-119	1:200
CD31	eFluor 450	eBioscience	390	1:400
CD140a	APC	eBioscience	APA5	1:100
CD140b	Biotin	eBioscience	APB5	1:100
Sca-1	FITC	eBioscience	D7	1:200
CD105	APC	eBioscience	MJ7/18	1:200
CD51	Biotin	Biolegend	RMV-7	1:100
CD90.2	FITC	eBioscience	30-H12	1:200
Streptavidin	APC eFluor 780	eBioscience	47-4317	1:400
Streptavidin	PE	eBioscience	12-4317	1:400

Table S2: Primers used for real time PCR.

Gene	Forward (5'-3')	Reverse (5'-3')
Dlx5	GCCCCTACCACCAGTACG	TCACCATCCTCACCTCTGG
Noggin	CACTATCTACACATCCGCCCAG	AGCGTCTCGTTCAGATCCTTCT
Id1	CTCTACGACATGAACGGCTGT	TGCTCACCTTGCGGTTCT G
Bmpr1a	GGTTCAGCGAACTATTGCCAAA	TCACCACGCCATTTACCCA
Bmpr2	TACAACACCACTCAGTCCGC	CCTGTCTCCTGTCAACATTCTG
Bmp2	TGGAAGTGGCCCATTTAGAG	TGACGCTTTTCTCGTTTGTG
Gapdh	AGGTCGGTGTGAACGGATTTG	TGTAGACCATGTAGTTGAGGTCA

Taqman primers

Gene	Assay reference
Gapdh	Mm99999915_g1
Bone sialoprotein	Mm00492555_m1
Osteocalcin	Mm03413826_mH
Alp	Mm01187117_m1

Table S3: Antibodies used for western blots.

Antibody	Source	Dilution	Company	Catalog#
Phospho-p44/42MAPK(ERK1/2)	Rabbit (poly-IgG)	1:1000	Cell signaling	9101
p44/42MAPK(ERK1/2)	Rabbit (poly-IgG)	1:1000	Cell signaling	9102
Phospho-AKT (Ser473)	Rabbit (poly-IgG)	1:1000	Cell signaling	9271
AKT(pan) (40D4)	Mouse (Mono-IgG)	1:1000	Cell signaling	2920
Phospho-Smad1/Smad5/ Smad9 (D5B10)	Rabbit (Mono-IgG)	1:1000	Cell signaling	13820
Smad1 (D59D7)	Rabbit (Mono-IgG)	1:1000	Cell signaling	6944
Phospho-Tyrosine(p-Try-100)	Mouse (Mono-IgG)	1:1000	Cell signaling	9411
PDGFRβ	Rabbit (mono-IgG)	1:1000	Thermo Scientific	MA5-15143
GAPDH	Rabbit (poly-IgG)	1:1000	Santa Cruz	Sc-25778
